# Antimicrobial Photodynamic Activity of the Zn(II) Phthalocyanine RLP068/Cl Versus Antimicrobial-Resistant Priority Pathogens

**DOI:** 10.3390/ijms26157545

**Published:** 2025-08-05

**Authors:** Ilaria Baccani, Sara Cuffari, Francesco Giuliani, Gian Maria Rossolini, Simona Pollini

**Affiliations:** 1 Department of Experimental and Clinical Medicine (DMSC), University of Florence, Largo Brambilla, 3, 50134 Florence, FI, Italy; ilaria.baccani@unifi.it (I.B.); sara.cuffari@unifi.it (S.C.); gianmaria.rossolini@unifi.it (G.M.R.); 2 Microbiology and Virology Unit, Careggi University Hospital, Largo Brambilla, 3, 50134 Florence, FI, Italy; 3 L. Molteni & C. dei Fratelli Alitti, Società di esercizio S.P.A. SS57 Loc. granatieri, 50018 Scandicci, FI, Italy; f.giuliani@moltenifarma.it

**Keywords:** multidrug-resistant pathogens, Zn(II) phthalocyanine, photoinactivation

## Abstract

The emergence and spread of antimicrobial resistance among pathogens are significantly reducing available therapeutic options, highlighting the urgent need for novel and complementary treatment strategies. Antimicrobial photodynamic therapy (aPDT) is a promising alternative approach that can overcome antimicrobial resistance through a multitarget mechanism of action, exerting direct bactericidal and fungicidal effects with minimal risk of resistance development. Although aPDT has shown efficacy against a variety of pathogens, data on its activity against large collections of clinical multidrug-resistant strains are still limited. In this study, we assessed the antimicrobial activity of the photosensitizer RLP068/Cl combined with a red light-emitting LED source at 630 nm (Molteni Farmaceutici, Italy) against a large panel of Gram-negative and Gram-positive bacterial strains harboring relevant resistance traits and *Candida* species. Our results demonstrated the significant microbicidal activity of RLP068/Cl against all of the tested strains regardless of their resistance phenotype, with particularly prominent activity against Gram-positive bacteria (range of bactericidal concentrations 0.05–0.1 µM), which required significantly lower exposure to photosensitizer compared to *Candida* and Gram-negative species (range 5–20 µM). Overall, these findings support the potential use of RLP068/Cl-mediated aPDT as an effective therapeutic strategy for the management of localized infections caused by MDR organisms, particularly when conventional therapeutic options are limited.

## 1. Introduction

Over the past decades, antimicrobial resistance (AMR) has emerged as one of the most critical global health challenges of the 21st century, threatening the effectiveness of traditional antibiotics. The overuse and misuse of antimicrobial agents in healthcare, agriculture, and animal husbandry, combined with the propensity for genome plasticity and acquisition of mobile genetic elements by various pathogens, have led to the increased and rapid emergence of resistant strains. This has led to increased morbidity and mortality rates and higher healthcare-associated costs [1].

AMR is particularly concerning in healthcare settings, where multidrug-resistant (MDR) Gram-positive cocci (e.g., *staphylococci* and *enterococci*), as well as MDR and pan-drug-resistant (PDR) *Enterobacterales* (e.g., carbapenem-resistant *Klebsiella pneumoniae*), and non-fermenting Gram-negative species (e.g., MDR *Pseudomonas aeruginosa* and PDR *Acinetobacter baumannii*) represent major pathogens. These microorganisms exert various resistance strategies, including efflux pumps, target mutations, and enzymatic inactivation of antibiotics, and they are frequently able to produce biofilm [1].

Moreover, resistance to conventional treatments among fungal pathogens is emerging as a significant and persistent threat, despite limited attention until recently [2]. In particular, pathogenic *Candida* species exhibit both intrinsic and acquired resistance mechanisms to various antifungal agents and are now regarded as highly successful fungal pathogens [3]. Notably, the recently described *Candida auris* has emerged globally as a multidrug-resistant healthcare-associated fungal pathogen responsible for severe nosocomial outbreaks worldwide [4].

In this scenario, novel and alternative treatment strategies are urgently needed [5]. Antimicrobial photodynamic therapy (aPDT) offers a promising alternative to traditional antibiotics. aPDT has been shown to effectively inactivate a wide range of bacterial pathogens, including multidrug-resistant strains and biofilm formers [6,7]. This therapeutic approach is based on three main components: a non-toxic dye known as a photosensitizer (PS), light at a specific wavelength to activate it, and molecular oxygen [8]. Upon light activation, the PS enters an excited state and generates reactive oxygen species (ROS), particularly singlet oxygen, which causes rapid, localized damage to proteins, lipids, and nucleic acids, ultimately leading to cytotoxic effects [8]. In bacteria, it is well established that the main targets of aPDT are the outer cell structures, such as the cytoplasmic membrane and cell wall, and that the PS does not act on a specific intracellular target, unlike conventional antibiotics [9]. At present, no conclusive data regarding the selection of resistant mutants to aPDT are available and some reports have described bacterial tolerance to PSs [10]. Nevertheless, the emergence and spread of resistance to aPDT appear to be unlikely [9,11,12].

RLP068/Cl is a Zn(II) phthalocyanine photosensitizer whose antimicrobial activity has been investigated in vitro against bacterial and fungal pathogens growing in planktonic and biofilm forms [12,13,14]. Its activity has also been evaluated in vivo in animal models and in observational studies for the treatment of chronic wounds [15,16,17,18]. In the latter cases, the CE-marked medical device VULNOFAST^®^ plus, which includes RLP068/Cl as the active component, was used for the topical photodynamic treatment of skin lesions and ulcers colonized by pathogenic microorganisms.

Although the antimicrobial activity of RLP068/Cl has been demonstrated against various pathogens [12,13], comprehensive in vitro studies on large collections of MDR strains remain scarce. Here, we present the in vitro antimicrobial effects of RLP068/Cl activated by a light-emitting diode (LED) light source emitting red visible light at 630 nm against a collection of MDR and PDR bacterial pathogens, as well as different *Candida* species. These results are intended to reinforce the clinical potential of an RLP068/Cl-based photodynamic therapeutic approach, offering deeper insight into its broad-spectrum efficacy against highly resistant microorganisms.

## 2. Results

The study evaluated the in vitro antimicrobial activity of RLP068/Cl, a novel patented tetracationic Zn(II) phthalocyanine chloride supplied by Molteni Farmaceutici, combined with an LED light source emitting red visible light at 630 nm. Antimicrobial activity was assessed against a panel of 49 MDR and PDR bacterial pathogens (Table 1 and Table 2) and of 10 *Candida* spp. strains (Table 3). The selected strains represent recent Italian epidemiology and were selected according to their resistance phenotype and genotype. In particular, among Gram-negatives, we selected strains exhibiting resistance phenotypes to: (i) carbapenems (including KPC-, NDM-, VIM-, IMP-, FIM-, OXA-, GES-, NMC-, and IMI-type carbapenemase-producing strains), and (ii) colistin (including an *mcr-1.2* harboring strain) (Table 1).

Among Gram-positives, we selected strains exhibiting resistance phenotypes to: (i) oxazolidinones (including OptrA and Cfr producers), (ii) methicillin, (iii) daptomycin, and (iv) glycopeptides (including VanA and VanB producers) (Table 2).

*Candida* strains of clinical origin were also selected, including representatives of the most clinically relevant species and the emerging nosocomial fungal pathogen *C. auris* (Table 3).

Known relevant resistance phenotypes of the selected strains are specified, and when available, resistant traits and known resistance gene variants for each strain are shown in parentheses (Table 1, Table 2 and Table 3).

**Figure 1:**
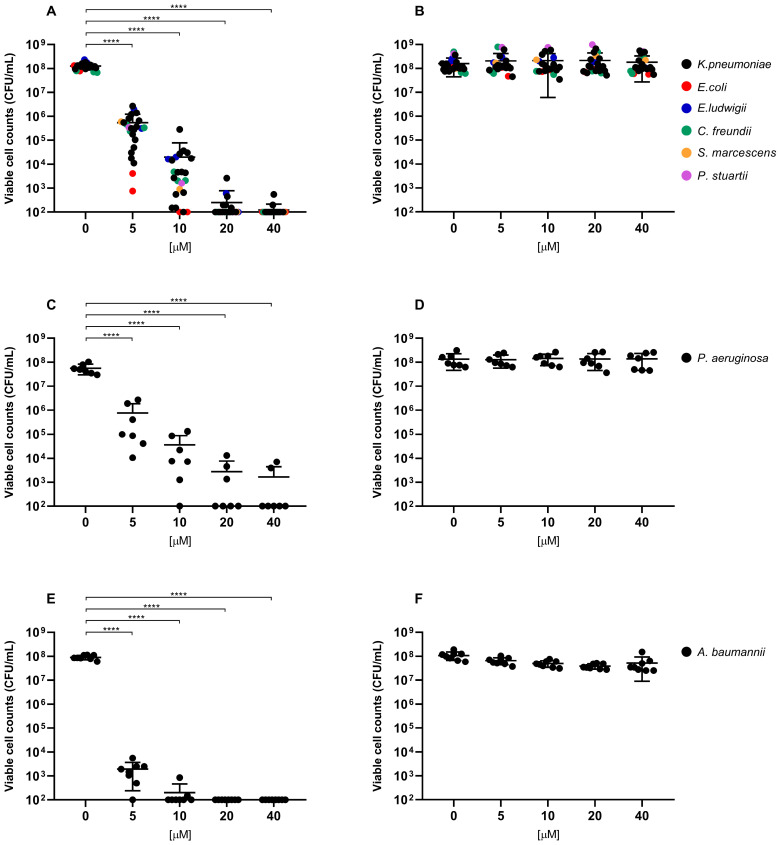
Antimicrobial activity of compound RLP068/Cl against Gram-negative strains (**A**,**B**: *Enterobacterales* strains, **C**,**D**: *P. aeruginosa* strains, **E**,**F**: *A. baumannii* strains). Graphs plot in log_10_ scale the means of replicates of viable cell counts (CFU/mL) for each strain and represent photoinactivation after exposure to RLP068/Cl and to a light source (**A**,**C**,**E** panels) or without light (**B**,**D**,**F** panels), respectively. Differences between means from strains exposed to different concentrations of RLP068/Cl and unexposed strains were statistically analyzed by one-way ANOVA test and significance has been reported on the graph for *p*-values < 0.05. ****: *p* < 0.0001.

**Figure 2:**
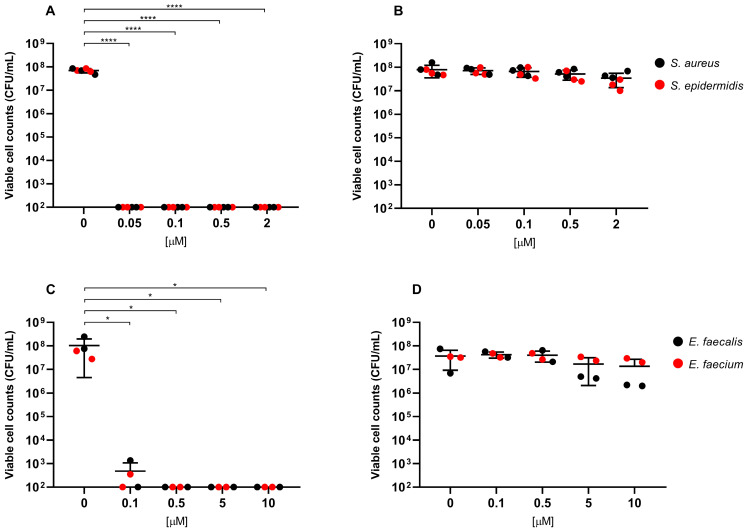
Antimicrobial activity of compound RLP068/Cl against Gram-positive strains (**A**,**B**: *Staphylococcus* species, **C**,**D**: *Enterococcus* species). Graphs plot in log_10_ scale the means of replicates of viable cell counts (CFU/mL) for each strain and represent photoinactivation after exposure to RLP068/Cl and to a light source (**A**,**C** panels) or without light (**B**,**D** panels), respectively. Differences between means from strains exposed to different concentrations of RLP068/Cl and unexposed strains were statistically analyzed by one-way ANOVA test and significance has been reported on the graph for *p*-values < 0.05. *: *p* < 0.05, ****: *p* < 0.0001.

**Figure 3:**
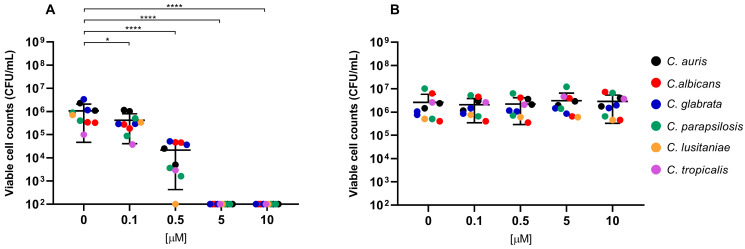
Antimicrobial activity of compound RLP068/Cl against *Candida* species. Graphs plot in log_10_ scale the means of replicates of viable cell counts (CFU/mL) for each strain. Photoinactivation after exposure to RLP068/Cl and to a light source or without light is represented in panel (**A**,**B**), respectively. Differences between means from strains exposed to different concentrations of RLP068/Cl and unexposed strains were statistically analyzed by one-way ANOVA test and significance has been reported on the graph for *p*-values < 0.05. *: *p* < 0.05, ****: *p* < 0.0001.

Overall, exposure to RLP068/Cl combined with red LED light resulted in a marked decrease in viable cells across all of the tested strains. CFU/mL reduction upon exposure was significant at all concentrations, with *p*-values ≤ 0.0001 in all cases except for *Candida* species tested with RLP068/Cl at a concentration of 0.1 μM (*p* ≤ 0.05). Experiments performed without light exposure showed that RLP068/Cl alone exerted minimal killing activity, confirming the absence of direct significant antimicrobial efficacy of the PS without light activation (Figure 1, Figure 2 and Figure 3, Supplementary Figures S1–S3). Some variability in susceptibility to aPDT was observed, particularly among *Klebsiella pneumoniae* and *Pseudomonas aeruginosa* isolates at lower PS concentrations (Figure 1 and Supplementary Figure S1). However, no significant difference correlated with strain-specific known resistance phenotypes or genotypes, possibly suggesting that other strain-dependent factors may influence photoinactivation efficiency.

Light-activated RLP068/Cl showed greater bactericidal activity against Gram-positive bacteria, requiring lower PS concentrations to achieve a 3-log reduction (MBC, ≥99.9% reduction in viable cell count) and 5-log reduction (≥99.999% reduction) in CFU/mL, compared to Gram-negative pathogens. MBC values for Gram-negatives mostly ranged from 5 to 10 μM, while Gram-positive species exhibited MBC values approximately 100-fold lower (range 0.05–0.1 μM) (Table 1 and Table 2).

Among Gram-positives, RLP068/Cl exhibited the highest antimicrobial activity against *Staphylococcus* spp. (including methicillin-resistant *S. aureus*), with an MBC and a 5-log reduction concentration less than or equal to 0.05 μM. *Enterococcus* spp. (including vancomycin-resistant *Enterococcus faecium* strains) required a slightly higher MBC (0.1 μM) than *Staphylococcus* spp. to achieve 3-log and 5-log reductions (Table 2).

Conversely, higher concentrations were needed to achieve a bactericidal effect among Gram-negative species. *Enterobacteriales* and *Pseudomonas aeruginosa* strains generally showed lower susceptibility to aPDT than *A. baumannii*. Indeed, MBC values for these strains ranged mostly from 5 to 10 μM, with some *P. aeruginosa* and *K. pneumoniae* strains exhibiting an MBC of 20 μM. Also, a 5-log reduction for the latter strains was only achieved at the highest tested PS concentration (40 μM) or could not be obtained in the tested experimental conditions. Conversely, *A. baumanii* strains showed relatively consistent susceptibility, with MBC values clustering between 5 and 10 μM.

The inactivation of *Candida* spp. was also evaluated, revealing a significant reduction in cell viability at PS concentrations less than or equal to 5 μM (*p* ≤ 0.0001) across all of the tested species. As also observed with Gram-negatives, susceptibility to aPDT varied among *Candida* species, with *C. lusitaniae* exhibiting higher susceptibility and an MBC of 0.5 μM.

## 3. Discussion

Over the last years, aPDT has emerged as a promising therapy to tackle antibiotic resistance and difficult-to-treat infections. Various PSs have been tested against different bacterial and fungal pathogens, showing significant reductions in viable cells in different test models, such as in vitro, in vivo, and biofilm models [15,19,20,21,22]. Nevertheless, the majority of existing data comes from type strains or small panels of clinical isolates [6,23,24,25,26]. Moreover, studies on phthalocyanine photosensitizers against clinical isolates are currently limited to a few representative species (e.g., *S. aureus*, *K. pneumoniae*, *P. aeruginosa*, *E. faecalis*, and *C. albicans*) [12,13,27,28,29].

In this study, we evaluated the antimicrobial efficacy of the photosensitized RLP068/Cl activated by 630 nm red light, against a large collection of clinical MDR/PDR strains representative of the most up-to-date resistance phenotypes and genotypes circulating in Italy’s territory and worldwide. To the best of our knowledge, this is the first comprehensive assessment of this PS’s antimicrobial activity against such a broad panel of resistant strains. For some bacterial species (e.g., some *Enterobacterales*, *Staphylococcus*, and *Enterococcus* spp.), only a small number of strains were included. Although no clear differences could be observed between different strains of these species, further studies could be performed to confirm the efficacy of RLP068/Cl against a more consistent number of strains.

The obtained results demonstrated significant photoinactivation efficiency for RLP068/Cl combined with light against all of the tested isolated. Particularly, Gram-positive bacteria required significantly lower concentrations of the molecule (range 0.05–01 µM) for a bactericidal effect compared to Gram-negative species (range 5–20 µM). These findings are overall consistent with results obtained using PSs sharing the same chemical structure and others belonging to different molecular classes [27,30,31]. The higher concentration of RLP068/Cl required to exert activity against Gram-negative pathogens likely reflects their intrinsic structural differences compared to Gram-positives. Indeed, Gram-positive bacteria have a thick but relatively porous peptidoglycan layer that allows easier penetration of PSs and ROS generated during aPDT. By contrast, Gram-negatives possess a more complex cell envelope with an outer membrane that acts as a barrier, limiting access of these compounds to the inner membrane and cytoplasm, thereby reducing aPDT efficacy [32,33,34].

Among tested Gram-positives, *staphylococci* were the most susceptible while *enterococci* showed, in our experiments, only slightly higher MBCs (0.05 vs. 0.1 μM). This finding aligns with available data reporting a substantial consistency of PS activity across both bacterial genera ([6] and references therein).

Among Gram-negative bacteria, *Pseudomonas aeruginosa* showed the lowest susceptibility, with some isolates requiring concentrations exceeding 40 μM for a 5-log reduction in viable cells. This observation is consistent with previous findings emphasizing the challenge of treating *P. aeruginosa* infections due to its efficient efflux system and altered membrane permeability, which also affect susceptibility to photodynamic therapy [35,36,37,38]. Indeed, efflux pumps actively expel photosensitizers from bacterial cells, thus reducing intracellular accumulation and aPDT effectiveness. Combining photosensitizers with efflux pump inhibitors has also been attempted to enhance bacterial susceptibility to aPDT by preventing PS expulsion and increasing reactive oxygen species-mediated inactivation [36,37].

The general susceptibility of *Candida* spp. to aPDT suggests that yeast cells may allow consistent uptake of RLP068/Cl. This result aligns with data reported by Souza et al. supporting that positively charged photosensitizers with higher lipophilicity exhibit enhanced cellular uptake in yeasts, improving photodynamic inactivation [39]. *C. lusitaniae* appears to be the most susceptible species; it differs from other *Candida* species in metabolic and structural features (e.g., mannans structure), which also influence antifungal susceptibility [40]. Whether these differences contribute to the increased susceptibility to aPDT remains unknown and requires further investigation.

To date, RLP068/Cl’s in vitro antibacterial activity was demonstrated against only a few strains of *S. aureus*, *P. aeruginosa* and *C. albicans*, with reports of remarkably different microbicidal activity toward different species [12,13]. Overall, our findings are consistent with those previously published, reporting MBCs in a comparable range of concentrations and confirming the higher susceptibility of Gram-positives with respect to Gram-negatives.

Overall, these findings demonstrate the effectiveness of RLP068/Cl as a photosensitizer for antimicrobial photoinactivation, particularly against Gram-positive bacteria and fungi. However, further investigation is needed to optimize treatment strategies for Gram-negative species, possibly by combining the PS with other membrane-active molecules, such as efflux pump inhibitors, or by modifying the PS molecule to enhance cellular uptake. Moreover, given its broad spectrum of activity, aPDT with RLP068/Cl could play a key role in treating polymicrobial infections, such as those in chronic wounds and sustained by biofilms, as reported also for other photosensitizers [14,41]. Indeed, RLP068/Cl’s anti-biofilm activity has been demonstrated in vitro against *P. aeruginosa* and *S. aureus* strains of clinical origin [14], but its efficacy against other biofilm-forming pathogens or polymicrobial biofilms remains to be studied. Moreover, several studies have also reported the clinical efficacy of aPDT with RLP068/Cl in vivo; in these cases, aPDT has been applied to chronic wounds of different types, which are often infected by a polymicrobial bacterial community that generally persist as a biofilm, resulting in reduced bacterial loads and clinical improvement [16,17,18].

## 4. Materials and Methods

### 4.1. Bacterial Strains and Culture Conditions

In total, 49 clinically relevant representatives of different *Enterobacterales*, non-fermenting Gram-negative and Gram-positive bacterial species, and 10 *Candida* spp. strains were selected for in vitro testing. The bacterial strain collection included clinically relevant multidrug-resistant (MDR) and pan-drug-resistant (PDR) phenotypes representative of recent Italian epidemiology (Table 1 and Table 2). Gram-negative strains included 15 *K. pneumoniae*, 2 *E. coli*, 3 *C. freundii*, 2 *E. ludwigii*, 1 *P. stuartii*, 1 *S. marcescens*, 7 *P. aeruginosa*, and 8 *A. baumannii* strains. Gram-positive strains included 3 *S. aureus*, 3 *S. epidermidis*, 2 *E. faecalis*, and 2 *E. faecium* strains. The *Candida* spp. panel included two reference strains of the emerging pathogen *C. auris* and 8 clinical strains of *C. albicans, C. glabrata, C. parapsilosis*, *C. lusitaniae*, and *C. tropicalis* (Table 3).

Bacterial strains were grown on Mueller Hinton Agar (MHA; Becton Dickinson Franklin Lakes, NJ, USA) or Tryptic Soy Agar (TSA; Oxoid, Thermo Fisher Scientific, Waltham, MA, USA) for *Enterococci* at 37 °C under aerobic conditions for 16–20 h unless otherwise specified.

*Candida* spp. strains were cultured on Sabouraud Agar (Oxoid, Thermo Fisher Scientific, Waltham, MA, USA) at 37 °C for 24–48 h unless otherwise specified.

### 4.2. Photosensitizer and Light Source

RLP068/Cl, a new patented tetracationic Zn(II) phthalocyanine chloride (MW 1320.52 g/mol) supplied by Molteni Farmaceutici (patent no. WO2011/012698 and no. WO2024/252344) and commercially available as a medical device (VULNOFAST^®^ plus, Molteni Farmaceutici), was used as the photosensitizer [42]. RLP068/Cl was prepared as a stock solution (1 mM) in dimethyl sulfoxide (DMSO; OriGen Biomedical, Austin, TX, USA); the stock solution was divided into aliquots and stored at −20 °C in the dark until used.

The illumination was carried out using a noncoherent LED lamp (VULNOLIGHT^®^, Molteni Farmaceutici, Italy) with a wavelength at 630 nm. The delivered light energy was 30 J cm^−2^, resulting from 4 min illumination at 120 mW cm^−2^.

### 4.3. Phototoxicity Assay

To perform the phototoxicity assay, RLP068/Cl was diluted in sterile PBS (Euroclone, Milan, Italy) with 10% DMSO to a concentration double of that needed for bacterial or yeast inactivation and was aliquoted into wells of a microtiter 96-well plate (50 μL/well).

A bacterial suspension was then prepared from single colonies of each strain in Dulbecco’s modified phosphate-buffered saline (PBS), pH 7.4, to an OD_600_ of ca 0.4–0.5. Bacterial suspensions were then plated into a microtiter 96-well plate (50 µL/well) to a final concentration of 5 × 10^7^–2 × 10^8^ CFU/mL for bacteria (depending on the bacterial species) and 10^6^ CFU/mL for yeasts. Bacterial cell suspensions were incubated in the dark with the appropriate RLP068/Cl concentrations (5, 10, 20, and 40 µM for Gram-negative strains; 0.1, 0.5, 5, and 10 µM for *Enterococcus* spp. strains; and 0.05, 0.1, 0.5, and 2 µM for *Staphylococcus* spp. strains) at room temperature for 5 min, while yeast suspensions were incubated under the same experimental conditions for 1 h. All microbial samples were illuminated with a fluence rate of 120 mW cm^−2^ for 4 min, delivering a final energy dose of 30 J cm^−2^. This was in agreement with the experimental procedures previously published on RLP068/Cl [12,13].

The intrinsic microbicidal activity of the PS was tested for each strain using cells exposed to the different tested concentrations of RLP068/Cl without illumination. The phototoxicity of the light source was tested for each strain using cells exposed to illumination without RLP068/Cl. Cell viability was assayed by 10-fold serially diluting samples in PBS and by plating in triplicate on MHA, TSA (for *enterococci*), and Sabouraud Agar (for *Candida* spp.). After incubation of the plates at 37 °C for 18–24 h, the number of CFU/mL was counted and the minimum bactericidal concentration (MBC) or minimum fungicidal concentration (MFC), defined as the lowest concentration of RLP068/Cl that resulted in 3.0-log_10_ and 5.0-log_10_ cell reductions, was calculated. The limit of detection for this method was approximately 10^2^ CFU/mL. Strains were tested in the presence of the LED light source at least in duplicate in independent experiments.

### 4.4. Statistics

Data are represented in Figure 1, Figure 2 and Figure 3 as the means of viable cell counts from separate replicated experiments; bars presented in the graphs represent the standard deviation of the mean (SD). Differences between means from strains exposed to the PS compared with unexposed strains were statistically analyzed by one-way ANOVA test. A *p*-value < 0.05 was considered significant and is represented in the graphs.

## 5. Conclusions

The high antimicrobial efficacy of RLP068/Cl supports aPDT based on this PS as a promising non-antibiotic strategy to tackle infection sustained by MDR bacteria and *Candida* species by exploiting ROS-mediated microbial inactivation, potentially representing an effective alternative to conventional antimicrobial treatments.

As the global burden of AMR continues to rise, exploring and implementing innovative therapies like aPDT becomes increasingly vital. Further research to optimize photosensitizer formulations, improve targeted delivery, and expand clinical applications will be crucial for integrating aPDT into mainstream medical practice. With its unique mechanism of action, aPDT represents a promising advance in addressing the AMR crisis.

## Figures and Tables

**Table 1:** In vitro photoinactivation of Gram-negative strains with compound RLP068/Cl. Strain resistance phenotypes and genotypes (in brackets) are shown. Bactericidal concentration (≥3-log and ≥5-log reductions in viable cell count) of RLP068/Cl is reported.

Strain ID	Species	Resistance Phenotype (Genes) ^a^	MBC (μM) ≥ 3 log ^b,c^	MBC (μM) ≥ 5 log ^b,c^
FI-01	*Klebsiella pneumoniae*	CEF^R^, COL^R^, AG^R^, FLQ^R^, CARBA^R^ (*bla*_NDM_) (*bla*_NDM_) (*bla*_NDM_) (*bla*_NDM_)	5	10
FI-02	*Klebsiella pneumoniae*	CEF^R^, AG^R^, CARBA^R^ (*bla*_NDM_)	5	10
FI-03	*Klebsiella pneumoniae*	CEF^R^, AG^R^, FLQ^R^, CARBA^R^ (*bla*_NDM_)	5	10
FI-04	*Klebsiella pneumoniae*	CEF^R^, AG^R^, FLQ^R^, CARBA^R^ (*bla*_NDM_)	10	20
FI-05	*Klebsiella pneumoniae*	CEF^R^, COL^R^, FLQ^R^, CARBA^R^ (*mcr*-1.2; *bla*_KPC-3_)	5	10
FI-06	*Klebsiella pneumoniae*	CEF^R^, COL^R^, FLQ^R^ (*bla*_VIM-1_)	10	20
FI-07	*Klebsiella pneumoniae*	CEF^R^, COL^R^, AG^R^, FLQ^R^, CARBA^R^ (*bla*_KPC-2_)	10	20
FI-08	*Klebsiella pneumoniae*	CEF^R^, COL^R^, FLQ^R^, CARBA^R^ (*bla*_KPC-3_)	10	20
FI-09	*Klebsiella pneumoniae*	CEF^R^, COL^R^, FLQ^R^, CARBA^R^ (*bla*_KPC-3_)	10	20
FI-10	*Klebsiella pneumoniae*	CEF^R^, COL^R^, AG^R^, FLQ^R^, CARBA^R^ (*bla*_KPC-3_)	5	20
FI-11	*Klebsiella pneumoniae*	CEF^R^, FLQ^R^, CARBA^R^ (*bla*_KPC-2_)	5	10
FI-12	*Klebsiella pneumoniae*	CEF^R^, AG^R^, FLQ^R^, CARBA^R^ (*bla*_OXA-48-like_)	10	20
FI-14	*Klebsiella pneumoniae*	CEF^R^, COL^R^, FLQ^R^, CARBA^R^ (*bla*_KPC-3_)	20	40
FI-15	*Klebsiella pneumoniae*	CEF^R^, FLQ^R^ (*bla*_KPC-31_)	10	20
FI-20	*Klebsiella pneumoniae*	CEF^R^, FLQ^R^, CARBA^R^ (*bla*_KPC-3_)	10	20
FI-13	*Escherichia coli*	CEF^R^, FLQ^R^ (*bla*_OXA-48-like_)	5	5
FI-16	*Escherichia coli*	CEF^R^, AG^R^, FLQ^R^, CARBA^R^ (*bla*_KPC_*, bla*_VIM_)	5	10
FI-17	*Serratia marcescens*	CEF^R^, COL^R^, AG^R^, FLQ^R^, CARBA^R^ (*bla*_NDM_)	10	10
FI-18	*Citrobacter freundii*	CEF^R^, FLQ^R^, CARBA^R^ (*bla*_VIM_)	10	20
FI-21	*Citrobacter freundii*	FLQ^R^, CARBA^R^ (*bla*_VIM_)	10	20
FI-22	*Citrobacter freundii*	CEF^R^, FLQ^R^, CARBA^R^ (*bla*_KPC_*, bla*_VIM_)	10	20
FI-24	*Providencia stuartii*	CEF^R^, AG^R^, FLQ^R^, CARBA^R^ (*bla*_KPC_)	10	20
FI-32	*Enterobacter ludwigii*	CARBA^R^ (*bla*_NMC-A_)	10	20
FI-33	*Enterobacter ludwigii*	CARBA^R^ (*bla*_IMI-2_)	10	20
FI-23	*Pseudomonas aeruginosa*	FLQ^R^, CARBA^R^ (*bla*_IMP-13_)	20	>40
FI-25	*Pseudomonas aeruginosa*	FLQ^R^, CARBA^R^ (*bla*_VIM-1_)	20	>40
FI-26	*Pseudomonas aeruginosa*	AG^R^, FLQ^R^, CARBA^R^ (*bla*_VIM-2_)	10	20
FI-27	*Pseudomonas aeruginosa*	FLQ^R^, CARBA^R^ (*bla*_VIM-1_)	10	20
FI-28	*Pseudomonas aeruginosa*	AG^R^, FLQ^R^, CARBA^R^ (*bla*_VIM-2_)	10	20
FI-29	*Pseudomonas aeruginosa*	AG^R^, FLQ^R^, CARBA^R^ (*bla*_GES-5_)	5	10
FI-34	*Pseudomonas aeruginosa*	CEF^R^, AG^R^, FLQ^R^, CARBA^R^ (*bla*_FIM_)	10	40
FI-30	*Acinetobacter baumannii*	AG^R^, FLQ^R^, CARBA^R^ (*bla*_OXA-23_)	5	10
FI-31	*Acinetobacter baumannii*	FLQ^R^, CARBA^R^ (*bla*_NDM_)	5	5
FI-35	*Acinetobacter baumannii*	AG^R^, FLQ^R^, CARBA^R^	5	10
FI-36	*Acinetobacter baumannii*	AG^R^, FLQ^R^, CARBA^R^	5	10
FI-37	*Acinetobacter baumannii*	AG^R^, FLQ^R^, CARBA^R^	5	5
FI-38	*Acinetobacter baumannii*	AG^R^, FLQ^R^, CARBA^R^	5	5
FI-39	*Acinetobacter baumannii*	AG^R^, FLQ^R^, CARBA^R^	5	10
FI-40	*Acinetobacter baumannii*	AG^R^, FLQ^R^, CARBA^R^ (*bla*_OXA-23_)	5	10

^a^ CEF^R^: resistance to cephalosporins; AG^R^: resistance to aminoglycoside; FLQ^R^: resistance to fluoroquinolones; CARBA^R^: resistance to carbapenems; COL^R^: resistance to colistin. ^b^ MBC: minimum bactericidal concentration. ^c^ Reductions of ≥3 log and ≥5 log in CFU/mL.

**Table 2:** In vitro photoinactivation of Gram-positive strains with compound RLP068/Cl. Strain resistance phenotypes and genotypes (in brackets) are shown. Bactericidal concentration (≥3-log and ≥5-log reductions in viable cell count) of RLP068/Cl is reported.

Strain ID	Species	Resistance Phenotype (Genes) ^a^	MBC (μM) ≥ 3 log ^b,c^	MBC (μM) ≥ 5 log ^b,c^
FI-43	*Staphylococcus aureus*	ERY^R^, OXA^R^, LIN^R^ (*cfr*, *poxtA*)	0.05	0.05
FI-45	*Staphylococcus aureus*	ERY^R^, OXA^R^	0.05	0.05
FI-50	*Staphylococcus aureus*	OXA^R^*,* DAPTO^R^	0.05	0.05
FI-44	*Staphylococcus epidermidis*	ERY^R^, OXA^R^, LIN^R^ (*cfr*)	0.05	0.05
FI-46	*Staphylococcus epidermidis*	ERY^R^, OXA^R^*,* DAPTO^R^	0.05	0.05
FI-47	*Staphylococcus epidermidis*	OXA^R^, LIN^R^ (*cfr*)	0.05	0.05
FI-41	*Enterococcus faecalis*	ERY^R^, OXA^R^, LIN^R^ (*optrA*)	0.1	0.1
FI-42	*Enterococcus faecalis*	ERY^R^, OXA^R^, LIN^R^ (*optrA*)	0.1	0.1
FI-48	*Enterococcus faecium*	GLY^R^ (*vanA*)	0.1	0.1
FI-49	*Enterococcus faecium*	GLY^R^ (*vanB*)	0.1	0.1

^a^ ERY^R^: resistance to erythromycin; LIN^R^: resistance to linezolid; OXA^R^: resistance to oxacillin; DAPTO^R^: resistance to daptomycin; GLY^R^: resistance to glycopeptides. ^b^ MBC: minimum bactericidal concentration. ^c^ Reductions of ≥3 log and ≥5 log in CFU/mL.

**Table 3:** In vitro photoinactivation of *Candida* spp. strains with compound RLP068/Cl. Strain features are reported. Fungicidal concentration (≥3-log reduction in viable cell count) of RLP068/Cl is reported.

Strain ID	Species	Resistance Phenotype ^a^	MBC (μM) ≥ 3 log ^b,c^
FI-51	*Candida auris*	None (Reference type strain)	5
FI-52	*Candida auris*	None (Reference type strain)	5
FI-53	*Candida albicans*	None	5
FI-54	*Candida albicans*	AZL^R^, ECIN^R^	5
FI-55	*Candida glabrata*	None	5
FI-56	*Candida glabrata*	None	5
FI-57	*Candida parapsilosis*	None	5
FI-58	*Candida parapsilosis*	ECIN^R^	5
FI-59	*Candida lusitaniae*	None	0.5
FI-60	*Candida tropicalis*	None	5

^a^ AZL: resistance to azoles; ECIN: resistance to echinocandins. ^b^ MBC: minimum bactericidal concentration. ^c^ Reduction of ≥3 log in CFU/mL.

## Data Availability

The original contributions presented in this study are included in the article and in the supplementary material.

## Supplementary Materials

The following supporting information can be downloaded at: https://www.mdpi.com/article/10.3390/ijms26157545/s1.
