# Supplementary material for: Antimicrobial Photodynamic Activity of the Zn(II) Phthalocyanine RLP068/Cl Versus Antimicrobial-Resistant Priority Pathogens
Source: Int J Mol Sci. 2025 Aug 5;26(15):7545. doi: 10.3390/ijms26157545 (PMC12347331; doi:10.3390/ijms26157545)
Supplement: Supplementary file 1 [file ijms-26-07545-s001.zip › ijms-3714497-supplementary.pdf]

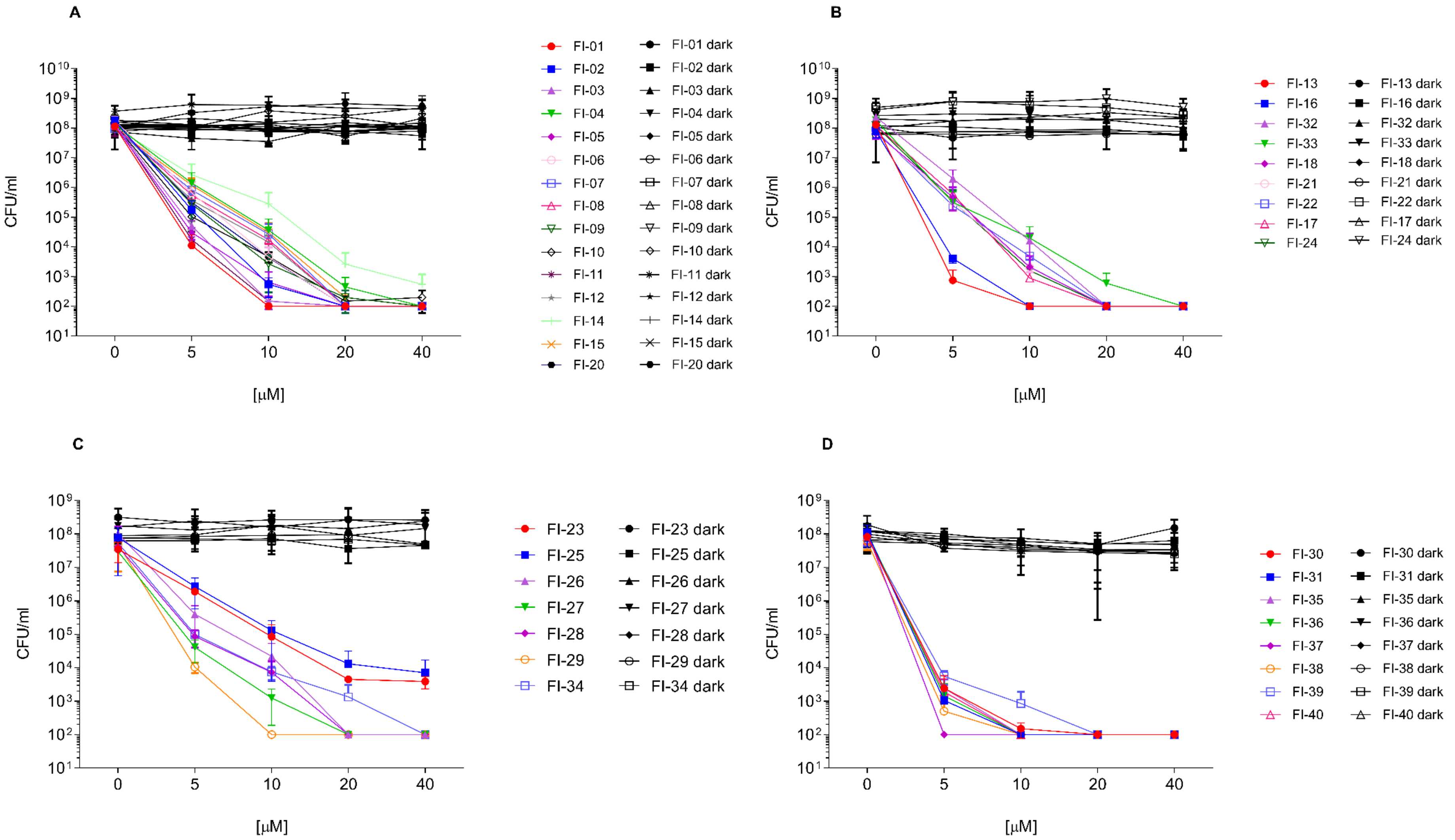

Supplemental Figure S1. Antimicrobial activity of compound RLP068/Cl on Gram-negative strains. Panel A: *Klebsiella pneumoniae* isolates; panel B: *Enterobacterales* strains other than *K. pneumoniae*; panel C: *P. aeruginosa* isolates; panel D: *A. baumannii* isolates. For strains' features, see Table 1. Graphs plot

replicates of viable cell counts for each strain and represent photoinactivation after exposure to RLP068/Cl and to a light source (coloured lines) or without light (black and grey lines).

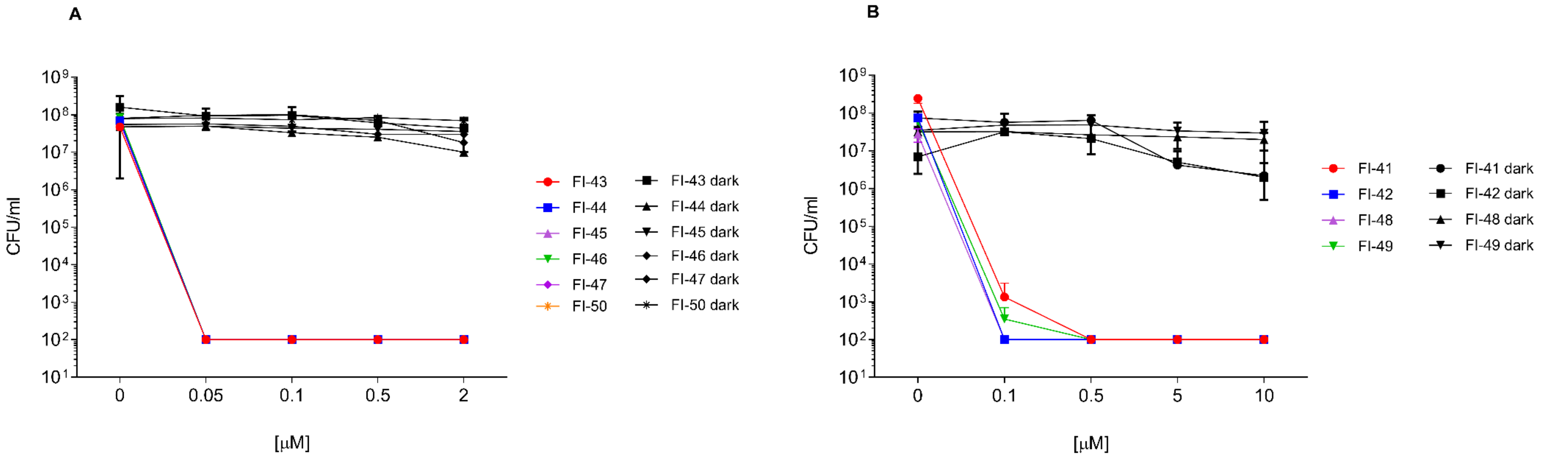

Supplemental Figure S2. Antimicrobial activity of compound RLP068/Cl on Gram-positive strains. Panel A: *Staphylococcus* spp. isolates; panel B: *Enterococcus* spp. isolates. For strains' features, see Table 2. Graphs plot replicates of viable cell counts for each strain and represent photoinactivation after exposure to RLP068/Cl and to a light source (coloured lines) or without light (black lines).

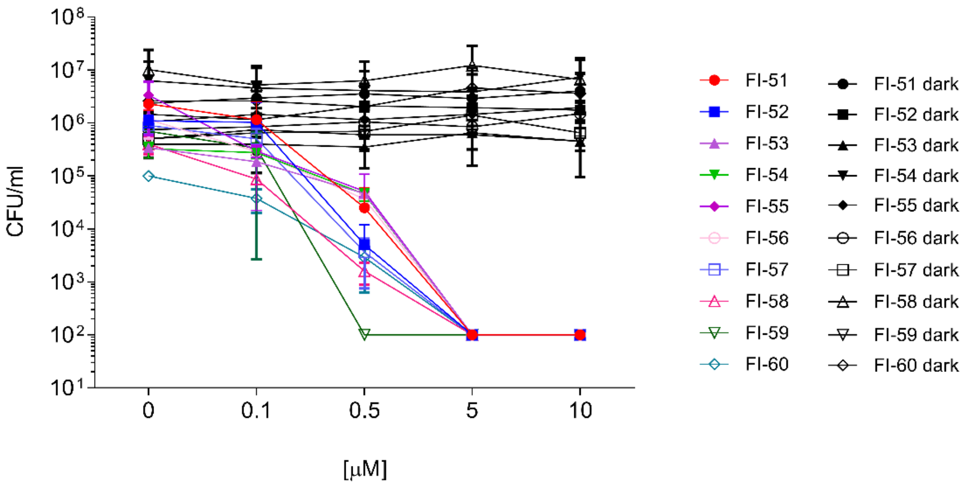

Supplemental Figure S3. Antimicrobial activity of compound RLP068/Cl on *Candida* spp. strains. For strains' features, see Table 3. Graphs plot replicates of viable cell counts for each strain and represent photoinactivation after exposure to RLP068/Cl and to a light source (coloured lines) or without light (black lines).
